# Preoperative sequential chemotherapy and hypofractionated radiotherapy combined with comprehensive surgical resection for high-risk soft tissue sarcomas: a retrospective study

**DOI:** 10.3389/fonc.2024.1423151

**Published:** 2024-06-19

**Authors:** Guoxin Qu, Zhichao Tian, Jiaqiang Wang, Chengliang Yang, Xiaohui Niu, Weitao Yao

**Affiliations:** ^1^ Department of Bone and Soft Tissue Cancer, The Affiliated Cancer Hospital of Zhengzhou University and Henan Cancer Hospital, Zhengzhou, China; ^2^ Department of Radiotherapy, The Affiliated Cancer Hospital of Zhengzhou University and Henan Cancer Hospital, Zhengzhou, China; ^3^ Department of Orthopedic Oncology Surgery, Beijing Ji Shui Tan Hospital, Beijing, China

**Keywords:** neoadjuvant chemotherapy, soft tissue sarcoma, sequential chemoradiotherapy, hypofractionated radiotherapy, high-risk soft

## Abstract

**Introduction:**

The management of soft tissue sarcomas presents considerable therapeutic challenges. This study was designed to assess the efficacy of neoadjuvant sequential chemotherapy and hypofractionated radiotherapy in conjunction with extensive surgical resection for the treatment of high-risk soft tissue sarcomas.

**Materials and methods:**

We performed a retrospective review of 31 high-risk soft tissue sarcoma patients treated at our institution from June 2021 to June 2023. The cohort consisted of 21 males and 10 females with a mean age of 55.7 years and included both initial and recurrent disease presentations. Our treatment regimen comprised two to three cycles of neoadjuvant chemotherapy coupled with hypofractionated radiotherapy, delivered at 5 Gy per fraction to a total dose of 25–35 Gy across 5–7 days, prior to surgical resection aimed at achieving wide margins. Data collection was systematic, covering surgical outcomes, chemoradiotherapy-related complications, and prognostic factors.

**Results:**

All patients completed the prescribed course of neoadjuvant chemoradiotherapy. 29% patients experienced grade 3+ chemotherapy toxicity, necessitating a reduction or interruption in their chemotherapy regimen. Limb preservation was accomplished in 30 patients finally. Response evaluation using RECIST 1.1 criteria post-neoadjuvant therapy revealed 9.7% with PD, 58.1% with SD, 29% with a PR, and 3.2% with a CR, culminating in an ORR of 32.2%. Postoperative complications included superficial wound infections in four patients and deep incisional infections in another four. 6 patients had developed metastasis, and 3 patients were still alive. Two experienced local recurrence. One-year DFS was 79.3%, with a one-year OS rate of 89.6%.

**Conclusion:**

Neoadjuvant sequential chemotherapy and hypofractionated radiotherapy followed by extensive surgical resection represents an effective treatment paradigm for high-risk soft tissue sarcomas. This multimodal approach not only facilitates tumor reduction but also significantly reduces the risks of local recurrence and distant metastasis.

## 1 Introduction

Soft tissue sarcomas (STS) represent a diverse group of mesenchymal-origin malignancies, with more than 100 different histologic subtypes according to the fourth edition of the World Health Organization (WHO) Classification of Tumours of Soft Tissue and Bone (1). Approximately 80% of STS cases arise in the extremities and trunk (2). These tumors exhibit a wide range of biological behaviors and are stratified into high, intermediate, and low-grade categories based on their aggressiveness. For low-grade STS, surgical resection remains the cornerstone of treatment. However, high-grade STS, notorious for aggressive local invasion and a propensity for distant metastasis, present therapeutic challenges, particularly in stages II and III, where the tumor burden is significant. Sole reliance on surgery in these cases increases the risk of local recurrence and may impede achieving clear surgical margins, especially when the tumor is in proximity to critical structures. Therefore, neoadjuvant therapies are required before surgical treatment (3).

Neoadjuvant therapies aim to improve local control of the tumor, prolong overall survival, and preserve limb function (4, 5). In extremity and trunk STS, both neoadjuvant and adjuvant radiotherapy have been shown to provide similar local control rates. However, neoadjuvant radiotherapy is associated with a higher incidence of wound complications postoperatively, whereas adjuvant radiotherapy carries an increased risk of chronic complications, such as fibrosis, joint stiffness, and lymphedema, which can significantly impair limb function (6–8). Research by Sampath et al. indicates potential survival benefits of preoperative neoadjuvant radiotherapy over postoperative adjuvant radiotherapy (7). The effectiveness and safety of adjuvant or neoadjuvant radiotherapy for soft tissue sarcomas is now unequivocal.

Numerous studies have explored the potential of adjuvant and neoadjuvant chemotherapy in the realm of localized STS. A seminal meta-analysis in 2008, which scrutinized 18 randomized controlled trials (RCTs), indicated that adjuvant chemotherapy could enhance disease-free survival (DFS), yet it did not correlate with an uptick in overall survival (OS) (9). Intriguingly, the perceived advantage of this treatment diminished with protracted follow-up in certain investigations. The EORTC 62931 trial, executed in 2012, was unable to substantiate a consistent benefit of adjuvant therapy using doxorubicin and ifosfamide across the entire patient spectrum; however, a demarcated subset of high-risk patients, as identified by a tailored nomogram, experienced positive outcomes (10). Subsequent research by the Italian Sarcoma Group in 2012 and 2016 corroborated these findings, demonstrating a DFS benefit from a concise course of adjuvant chemotherapy in patients at elevated risk (11). The ISG-STS 1001 trial, conducted from 2017 to 2020, further confirmed that histotype-tailored neoadjuvant chemotherapy markedly improved DFS when contrasted with standard chemotherapy protocols in high-risk categories, as defined by a nomogram (12). Recent studies advocate that chemotherapy could potentially ameliorate the prognosis for a specific subset of high-risk STS patients, especially those classified through prognostic nomograms.

Gobo Silva et al. found that neoadjuvant hyperfractionated radiotherapy combined with chemotherapy may increase the rates of limb preservation and is well-tolerated by patients. However, these findings are based on studies with small sample sizes and require further validation in larger patient cohorts (13). To verify the efficacy and safety of this protocol in more subtypes, our center has instituted an innovative preoperative therapeutic protocol for the treatment of high-risk soft tissue sarcomas. This protocol amalgamates sequential intensive fractionated radiotherapy with chemotherapy, culminating in surgical excision. The preliminary outcomes of this cohesive, multimodal strategy have been affirmative, as evidenced by our latest follow-up statistics, which signal a trend towards improved patient outcomes.

## 2 Materials and methods

### 2.1 Clinical data

We gathered information from patients diagnosed with trunk and soft tissue sarcomas who underwent surgical procedures at our hospital between June 2021 and June 2023, and follow-up was until June 2024.

### 2.2 Inclusion criteria

Patients classified as stage III according to the AJCC staging system for soft tissue sarcoma (14), or those at stage II with tumors in close proximity to significant vascular or nerve, situated within the deep fascial layer, and histopathologically classified as G3, are subjected to a multidisciplinary team (MDT) consultation at our institution prior to initiating treatment. The patient received neoadjuvant chemotherapy and hypofractionated radiotherapy. Patients presenting with primary or recurrent soft tissue sarcomas of the extremities or trunk.

### 2.3 Exclusion criteria

Preoperative examinations revealing lung metastasis, bone metastasis, or skip lesions. Palliative surgery with R1 or R2 margins. Loss to follow-up.

### 2.4 General information

From June 2021 to June 2023, our hospital treated a total of 31 patients who met the inclusion and exclusion criteria. Among them, 21 were males and 10 were females, with ages ranging from 27 to 71 years and an average age of 55.7 years.

### 2.5 Clinical and imaging presentation

All patients presented with a primary clinical manifestation of a localized mass, with 13 cases accompanied by pain, 16 cases with limb swelling, and 5 cases with functional impairment. Among them, 24 were initial diagnoses, and 7 were recurrent cases. The time from symptom onset to consultation ranged from 1 to 8 months, with an average of 3.5 months. All patients underwent preoperative magnetic.

### 2.6 Preoperative neoadjuvant therapy

The preoperative regimen involves the administration of 2–3 cycles of chemotherapy. For patients who have either previously received first-line chemotherapy and experienced a recurrence or are unable to tolerate the first-line regimen, second-line therapy options are explored. The initiation of neoadjuvant radiotherapy coincided with the administration of the final preoperative chemotherapy. The preoperative radiotherapy (RT) scheme consisted of 5 Gy per fraction for a total dose of 25–35Gy, with the aim of facilitating an R0 surgical resection.

For the RT planning, the gross tumor volume (GTV) was contoured on planning CT fused with contrast enhanced MRI or diagnostic contrast-enhanced CT. Clinical target volume (CTV) fully corresponded to GTV. Planning tumor volume (PTV) was created with 5 mm margins added to CTV. All patients were treated with IMRT (VMAT technique) plans generated with Eclipse (Varian) planning system version 5.1 using 6 MV X-rays. The VMAT plans were done with multiple arcs so that at least 95% of the PTV received 100% of the dose. In cases where the tumor was close to the skin, the skin flash tool was used (15, 16). Normal tissue constraints included: (1) Part of the longitudinal skin and subcutaneous tissue contralateral to the target area was protected as much as possible; (2) An uninvolved skin strip >2 cm in size coplanar to the PTV to <10 Gy mean dose; (3) ALARA principles were utilized for any joint in proximity to PTV; (4) If the dose to the PTV conflicts with the dose to the organs at risk, normal tissue restriction should be given priority.

### 2.7 Surgical procedure

Following completion of radiotherapy, all patients were scheduled for surgical resection within a period of 1 to 2 weeks. All surgical procedures were performed by skilled musculoskeletal surgeons after comprehensive preoperative planning. Adhering to the core principles of sarcoma surgery, the technique entailed a wide excision, which involved the complete removal of the tumor along with a margin of 3–5 cm of healthy muscle tissue surrounding it. In cases where tumors were located near vital vascular nerves, the associated vascular and nerve sheaths were also excised. Subperiosteal resection was conducted when the tumor was in close proximity to the bone membrane. If there was any suspicion of margin involvement during surgery, intraoperative frozen section analysis was utilized. Upon confirmation of margin involvement, immediate re-resection was performed. The cavity left after tumor excision was extensively irrigated with sterile distilled water for more than 10 minutes. In patients who had vascular nerves and bone membranes removed, the wound was bathed in anhydrous alcohol for over 10 minutes. Postoperatively, the wound cavity was properly drained, with the drainage tube being removed once the output decreased to less than 20 ml per day. Prophylactic antibiotics were prescribed for 24–48 hours, depending on the patient’s clinical status.

### 2.8 Postoperative rehabilitation and subsequent treatment

During the initial postoperative period, the affected limbs were appropriately immobilized and subjected to functional exercises. The gradual resumption of walking and usual limb activities began 2–4 weeks following the surgery. Additional chemotherapy commenced 2–3 weeks after the wound had sufficiently healed. Following routine pathological examinations, if the margins were classified as R1 or if there was intraoperative evidence of invasion into vital vascular nerve sheaths, adjuvant postoperative radiotherapy was implemented. If the wound heals well after surgery, adjuvant radiotherapy would be started within 6–8 weeks after surgery. The postoperative irradiation was performed as 25 fractions of 2 Gy. Preoperative MRI, surgical and pathology reports were used to create postopGTV. PostopCTV included postopGTV with 3 cm margins in cranio-caudal direction and 1.5 cm margins in the radial dimensions (without going beyond the boundaries of the involved compartment) (15).

### 2.9 Postoperative follow-up

Regular follow-up appointments were scheduled after surgery. For the first two years, follow-ups occurred every three months. From the third to the fifth year, the visits were scheduled semi-annually, and after five years, annual follow-ups were established. Follow-up evaluations included chest CT (computed tomography) scans, MRI (magnetic resonance imaging) of the operative site, and abdominal ultrasound to assess the liver, gallbladder, pancreas, and spleen. Patients with a risk of lymph node metastasis were monitored with routine regional lymph node ultrasound exams.

### 2.10 Data collection and statistical analysis

Data collection comprehensively covered patient demographics such as gender, age, and disease characteristics, as well as therapeutic interventions including radiotherapy, chemotherapy, and any complications arising from surgery.

The pathological response was assessed according to the criteria of the European Organization for Research and Treatment of Cancer-Soft Tissue and Bone Sarcoma Group (EORTC-STBSG) (17). The categories are defined as follows: (A) no stainable tumor cells; (B) single stainable tumor cells or small clusters (overall below 1% of the whole specimen); (C) >1%-<10% stainable tumor cells; (D) >10%-<50% stainable tumor cells; (E) >50% stainable tumor cells. The surgical specimens were reviewed by a pathologist and near pathological complete response (pCR) was defined as greater than or equal to 95% regressive changes (or less than 5% residual visible tumor cells) (18, 19).

The chemotherapy toxicities were assessed according to the Common Terminology Criteria for Adverse Events version 5.0 (CTCAE 5.0). Significant chemotherapy toxicity was defined as toxicity that caused a dose reduction or interruption in chemotherapy. The Kaplan-Meier method was employed to estimate both overall survival and disease-free survival rates. Statistical significance was established at a threshold of α=0.05, with P values below this cutoff considered significant. For the statistical analysis, SPSS software (version 20.0) was utilized.

## 3 Results

### 3.1 Patient demographics and clinical characteristics

This study encompassed a cohort of 31 patients who satisfied the inclusion criteria, comprising a diverse array of soft tissue sarcoma subtypes: 7 patients with undifferentiated pleomorphic sarcoma, 5 with myxoid fibrosarcoma, 3 with Ewings sarcomas, 2 with synovial sarcoma, 2 with rhabdomyosarcoma, 1 with CIC-rearranged sarcoma, 1 with liposarcoma, 1 with epithelioid sarcoma, and 9 patients presenting with sarcomas of indeterminate subtype. Among these, 24 were obtained from patients at initial diagnosis and the remaining 7 samples from relapsed patients. According to the stages of AJCC soft tissue sarcoma, there were 3 cases of stage II, 15 cases of stage IIIA, and 13 cases of stage IIIB (**Table 1**).

**Table 1:** Clinicopathologic feature.

Patient characteristics		Value
Gender, n (%)	Male	21 (67.7)
	Female	10 (67.7)
Age, years	Mean (range)	55.7 (27–71)
Location, n (%)	Upper limbs	4 (12.9)
	Trunk	11 (35.5)
	Lower limbs	16 (51.6)
Stage, n (%)	II	3 (51.6)
	IIIA	15 (48.4)
	IIIB	13 (41.9)
Pathological subtype, n (%)	undifferentiated pleomorphic sarcoma	7 (22.6)
	Myxoid fibrosarcoma	5 (16.1)
	synovial sarcoma	2 (6.5)
	rhabdomyosarcoma	2 (6.5)
	Ewings sarcomas	3 (9.7)
	CIC-rearranged sarcoma	1 (3.2)
	liposarcoma	1 (3.2)
	epithelioid sarcoma	1 (3.2)
	indeterminate subtype	9 (29)
Largest tumor dimension, cm (range)	mean	10.8 (4–24)
Type of patient, n (%)	initial diagnosis	24 (77.4)
	relapsed patients	7 (22.6)
First-line chemotherapy, n (%)	2 cycles	20 (64.5)
	3 cycles	7 (22.6)
Radiation dose, n (%)	25Gy	19 (61.3)
	30Gy	1 (3.2)
	35Gy	11 (35.5)

### 3.2 Neoadjuvant therapy and clinical result

In our cohort, one patient with a recurrence of epithelioid sarcoma, having not achieved remission with initial anthracycline-based chemotherapy, was administered second-line therapy centered around paclitaxel. Nine patients experienced grade 3+ chemotherapy toxicity, necessitating a reduction or interruption in their chemotherapy regimen. Among these individuals, three elderly patients who exhibited intolerance to first-line neoadjuvant chemotherapy were transitioned to anlotinib capsules as second-line therapy following one cycle. Subsequently, radiotherapy and surgery were performed after two cycles, deviating from the standard treatment protocol. The majority, comprising 27 patients, received first-line neoadjuvant chemotherapy, either as monotherapy or in combination, as determined by the multidisciplinary team (MDT) to tailor the treatment to individual patient needs.

Within this subset, the remaining 27 patients, dose adjustments were necessitated for six individuals due to bone marrow suppression or other adverse reactions, while 20 patients were able to complete the intended two cycles of neoadjuvant chemotherapy, and 7 patients managed to complete three cycles.

Regarding preoperative radiotherapy, the standard fraction size was 5 Gy. Nineteen patients received a total dose of 25 Gy, one patient was administered 30 Gy, and 11 patients received a higher cumulative dose of 35 Gy. Postoperatively, five patients were selected for additional adjuvant radiotherapy to manage their disease more aggressively.

Prior to the initiation of chemoradiotherapy, tumor dimensions were meticulously measured, revealing a range in size from 4 cm to 24 cm and an average diameter of 10.8 cm. Notably, following radiotherapy, 11 patients displayed an increase in tumor size. Of particular clinical interest was the subset of 3 patients with stable disease who, despite the lack of dimensional progression, exhibited significant volumetric growth on MRI scans, indicative of extensive liquefactive necrosis. These MRI observations were later substantiated by postoperative pathological examination, which confirmed the presence of substantial tumor necrosis (**Table 1**).

### 3.3 Surgical information

The 31 patients cohort underwent successful limb-salvage surgeries. In 12 cases, precise dissection techniques were imperative to isolate and resect tumor-involved vascular and neural structures. The volume of intraoperative blood loss varied, ranging from a minimum of 50 mL to a maximum of 2000 mL, with an average loss of 320 mL. The duration of the surgical procedures also varied, from as short as 40 minutes to as long as 180 minutes, with an average time of 68 minutes. R0 resection was performed in 29 patients and R1 resection in 2 patients. Twenty patients underwent straightforward tumorectomies. 6 patients used pedicled axial flaps to repair wounds because of large skin defects. In 5 patients, the pedicled muscle flap technique was used to fill the wound cavity due to the large size of the wound cavity and the exposed blood vessels and nerves.

### 3.4 Pathologic response

The pathological response was grade A in 2 patients (6.4%), grade B in 7 patients (22.6%), grade C in 10 patients (32.2%), grade D in 6 patients (19.4%), and grade E in 6 patients (19.4%). Near pCR were observed in 10 patients (32%).

### 3.5 Postoperative complications and management

**Figure 1:**
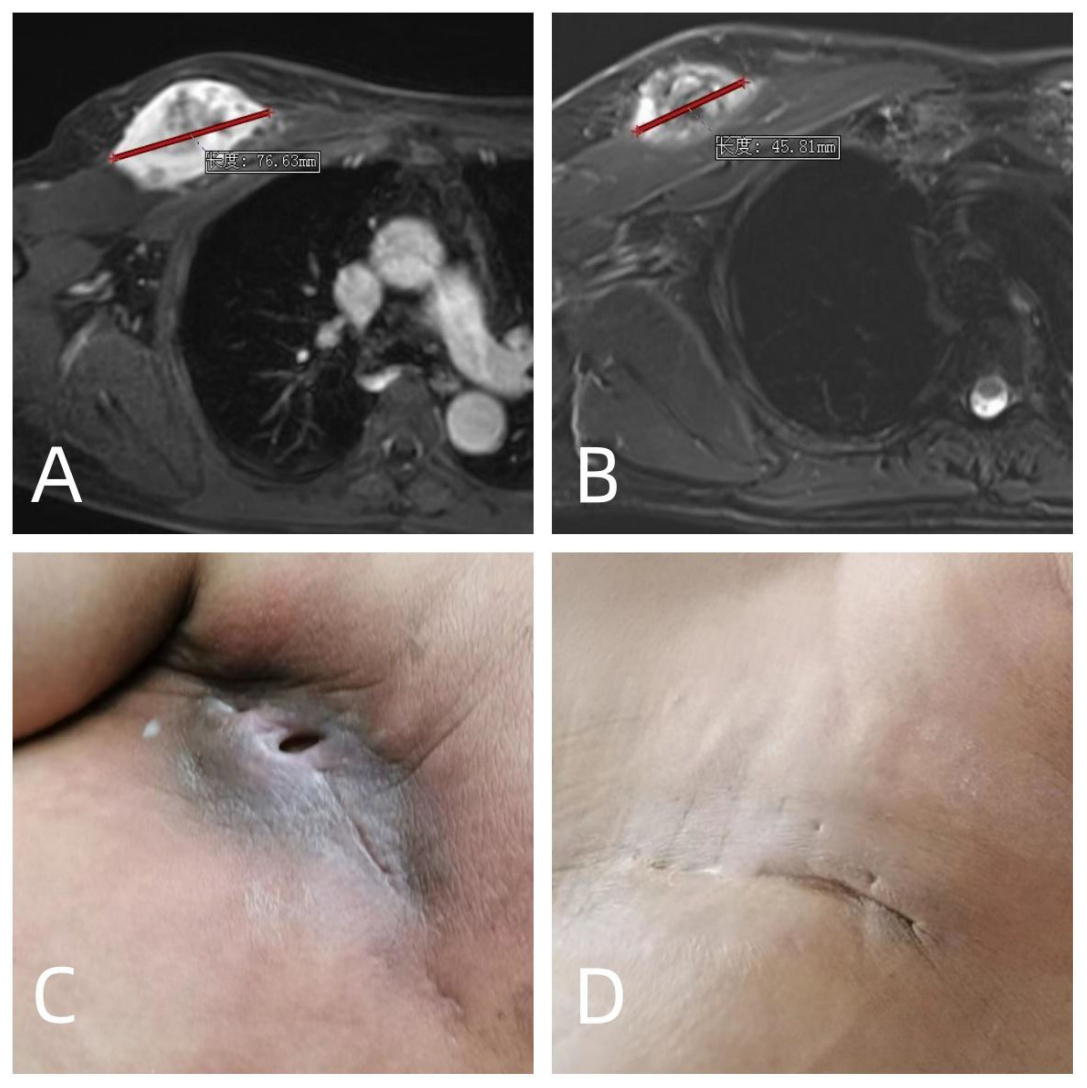
A 58 years old male who diagnosed with soft tissue sarcoma T2N0M0G3 IIIA in the right chest wall was treated with 2 cycles of preoperative doxorubicin and ifosfamide, neoadjuvant radiotherapy 35Gy, and surgery 13 days after neoadjuvant radiotherapy. **(A)** MRI before neoadjuvant chemoradiotherapy; **(B)** MRI after neoadjuvant chemoradiotherapy, which were evaluated as PR according to RECIST1.1 criteria; **(C)** long-term poor wound healing with pain after operation; **(D)** The patient underwent a second operation 3 months after the first operation. The necrotic area and infected area of the original radiotherapy were cleared, and the pain and infection were cured.

The postoperative period was characterized by a range of complications. Seroma formation was observed in sixteen patients, with durations ranging from three months to two years. The majority of these cases were managed with conservative measures, such as compression garments and close monitoring. Despite this, six patients required interventional drainage to resolve the fluid accumulations and promote healing. In four instances, superficial surgical site infections compromised wound integrity, necessitating aggressive local care including antibiotic therapy and surgical debridement in some cases. Furthermore, four patients suffered from deep tissue infections, with one patient experiencing significant pain due to the infection. This patient’s pain and infection were markedly improved following a secondary operative procedure to debride the infected and necrotic tissue, a complication likely exacerbated by prior radiotherapy. Another patient with deep soft-tissue infections presented and persistent stiffness in the knee joint, which remained unresolved at the 7-month postoperative mark, indicating a potential need for further intervention or long-term physical therapy to restore joint function and wound healing. Additionally, one patient exhibited signs of cutaneous sclerosis, a known late effect of radiotherapy (**Figure 1**).

### 3.6 Follow-up and survival outcomes

All patients underwent comprehensive postoperative surveillance, with follow-up periods ranging from 11 to35 months and an average follow-up of 20 months. 9 patients were monitored for over two years, while 29 patients had follow-ups exceeding one year.

Within the follow-up, a patient with a history of recurrent soft tissue sarcoma in the forearm encountered a second recurrence five months after surgery. This recurrence was decisively treated with an above-elbow amputation. Another recurrent patient relapsed 27 months after surgery and the tumor was unresectable. She was now receiving comprehensive treatment. Additionally, two patients developed postoperative pulmonary metastases, with one being the patient with recurrent forearm sarcoma. One patient with pulmonary metastases passed away 6 months post-detection, and the other died 8 months later. One patient was diagnosed with axillary lymph node metastasis in the drainage area 15 months after surgery. Three patients presented with multiple systemic metastases, and one of whom died 5 months post-detection. At present, 6 patients had developed metastasis, and 3 patients were still alive.

**Figure 2:**
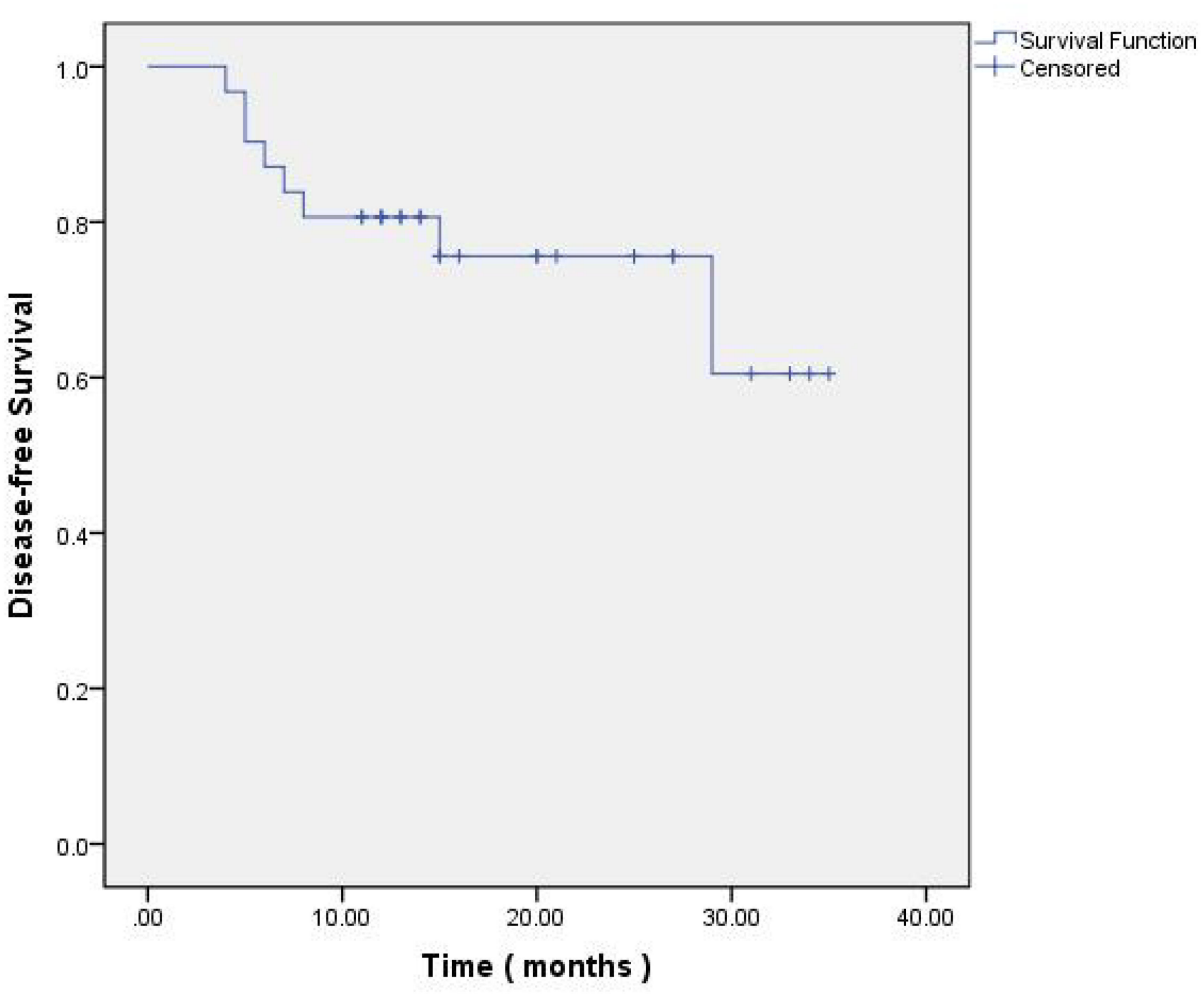
Kaplan-Meier of Disease-free Survival.

**Figure 3:**
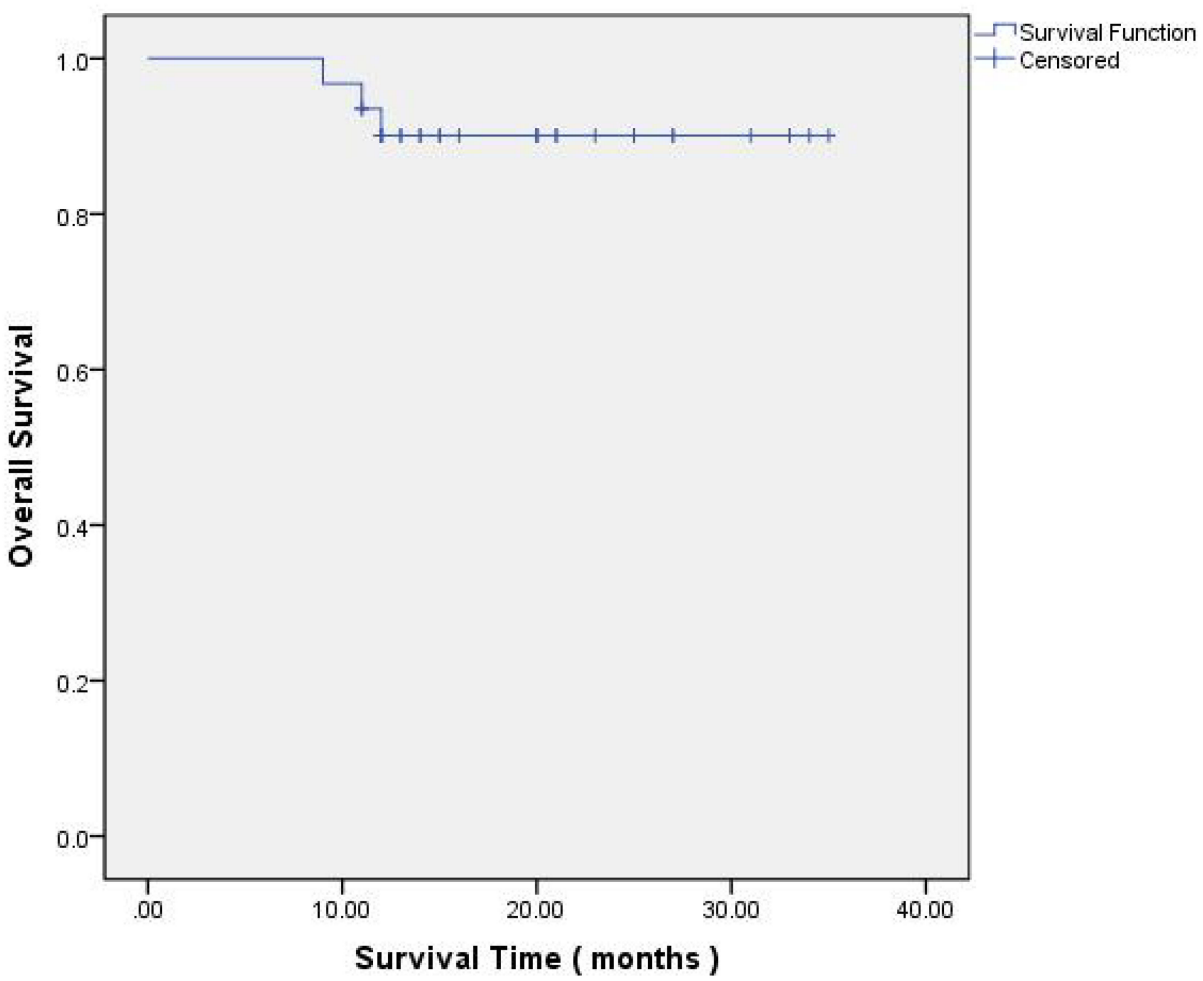
Kaplan–Meier of Overall Survival.

The post-treatment outcomes, evaluated according to the Response Evaluation Criteria in Solid Tumors (RECIST) version 1.1 (20), were as follows: Progressive Disease (PD) was observed in 9.7% (3/31) of cases, Stable Disease (SD) in 58.1% (18/31), Partial Response (PR) in 29% (9/31), and Complete Response (CR) in 3.2% (1/31). The Overall Response Rate (ORR) was 32.2% (10/31 cases). The Disease-Free Survival (DFS) rate of one year stood at 79.3% (23/29 cases), and the Overall Survival (OS) rate of one years was 79.3% (23/29 cases) at the time of this analysis. These outcomes are further elaborated in **Table 2** and depicted in **Figures 2**, **3**.

**Table 2:** Efficacy, complications and prognosis.

		Value
Effect of neoadjuvant therapy, n(%)	PD	3(9.7)
	SD	18(58.1)
	PR	9(29)
	CR	1(3.2)
	ORR	10(32.2)
Chemotherapy toxicity, n(%)	Grade 3+	9(29)
Postoperative complication, n(%)	Superficial tissue infection	4(12.9)
	Deep tissue infections	4(12.9)
	Cutaneous sclerosis	1(3.2)
	ankylosis	1(3.2)
Postoperative follow-up time, months	Mean ((range))	20(11–35)
	≥24	9(29)
	≥12	29(93.5)
Prognosis, n(%)	local recurrence	2(6.4)
	distant metastasis	6(19)
	OS of one year	26/29(89.6)
	DFS of one year	23/29(79.3)

PD, Progression of Disease; SD, Stable Disease; PR, Partial Response; CR, Complete Response; ORR, Objective Response Rate; OS, Overall Survival; DFS, Disease-free Survival.

## 4 Discussion

High-risk soft tissue sarcomas are indeed a significant clinical challenge due to their aggressive nature. These tumors are characterized by rapid growth rates, a high degree of malignancy, and complex anatomical locations that often make achieving complete surgical resection difficult (21). Even when a macroscopically complete resection is achieved, microscopic residual disease may remain, which can lead to local recurrence (22). Furthermore, these tumors have a tendency to metastasize, most commonly to the lungs, and the overall prognosis is often poor, with a lower 5-year survival rate compared to low-grade sarcomas (1).

Radiotherapy stands as a cornerstone adjuvant modality in the management of sarcomas. Conventional preoperative radiotherapy, typified by the administration of low individual doses (commonly below 2 Gy) over a protracted period, has been known to precipitate pronounced edema in the early phase and fibrosis subsequently. Such intensified tissue reactions significantly exacerbate the complexity of subsequent surgical interventions, leading to increased tissue delicacy, substantial surgical wound bleeding, and severe tissue adhesion, which complicates anatomical dissection (6). These challenges can escalate to vascular rupture and intricate surgical repairs. In contrast, preoperative radiotherapy delivered in large segments can be completed expeditiously, with diminished early edema and inflammatory response. It is thus our recommendation to undertake the surgical excision within a fortnight of completing radiotherapy, aiming to reduce the radiotherapeutic impact on the surgical milieu (23). Moreover, surgical efforts should be concentrated on resecting areas exhibiting a radiation response, thereby averting complications such as deep incisional infections stemming from residual necrotic soft tissues, which have the potential to cause persistent wound non-union and chronic discomfort (24, 25).

The role of preoperative radiotherapy in the management of high-risk soft tissue sarcomas has been the subject of extensive research (26). The rationale behind preoperative radiotherapy is multifaceted. ①Tumor Cell Reduction. Radiotherapy can reduce the number and viability of tumor cells within the primary tumor mass. This can be particularly important in the context of the ‘false capsule,’ a layer of compressed fibrous tissue that surrounds many soft tissue sarcomas. Although it may appear as a capsule, it is not a true biological barrier and can contain microscopic extensions of the tumor. By reducing the tumor burden in this area, the risk of leaving behind residual disease during surgery is diminished (27). ②Management of Edema and Skip Lesions. The edematous regions surrounding the tumor may harbor dispersed tumor cells or ‘skip lesions,’ which are foci of tumor cells located within the same compartment as the primary tumor but separated from it by normal tissue. Preoperative radiotherapy can target these areas, potentially eliminating microscopic disease that might not be addressed by surgery alone. ③Recurrence Prevention. Studies have shown that preoperative radiotherapy can help to control postoperative recurrence. This is particularly significant for intermediate to high-risk soft tissue sarcomas, where the risk of recurrence is higher. By reducing the tumor size and treating potential microscopic disease, the likelihood of complete surgical resection is increased, and the risk of recurrence is decreased (28). ④Outcomes with R1 Resections. Interestingly, research has indicated that even when tumor margins remain positive after preoperative radiotherapy (R1 resection), the rates of postoperative local recurrence are lower than might be expected (29). This suggests that radiotherapy may have a sterilizing effect on the microscopic residual disease, thereby preventing the regrowth of the tumor.

For conventional fractionation preoperative RT, the prescription of 50 Gy in 1.8–2 Gy once-daily fractions over 5–6 weeks, is the current standard schedule (27). Limb-salvage surgery combined with conventional fractionation preoperative RT had resulted in local control rates of at least 85% to 90% in patients with soft tissue sarcomas of the extremities (6, 30). However, the risk of wound complication in conventional fractionation RT was about 35% (6, 18). Considering the same pathological criteria, the rates of near pCR after conventional preoperative RT were about 8–10% (31, 32). In hypofractionated radiotherapy, Guadagnolo et al. (33) reported wound complications in 38% of 120 patients, and 5% patients had developed a local relapse at a median time of 16 months. Leite et al. (16) reported 28% patients presented wound complications, and 13.4% patients needed reoperation. Leite et al. (16) and Spałek et al. (34) reported near pCR 20.8% and 40%, respectively. In our study, wound complication occurred in 25.8% of patients, and the near pCR was 32%. Preoperative hypofractionated RT compared with the conventional fraction, hypofractioned RT did not increase the incidence of complications of wound. Moreover, the pCR was significantly increased due to the larger single radiation dose. In our study, CTV fully corresponded to GTV, so probably the wound complications were lower. For the same chemoradiotherapy combined with neoadjuvant regimen, we performed surgery within 2 weeks after radiotherapy, so the pCR of contrast Spałek et al. was lower.

It’s imperative to acknowledge the common complications associated with preoperative radiotherapy, notably including an elevated incidence of wound-related complications and persistent postoperative pain within the surgical region (6). Within our study, the likelihood of postoperative wound necrosis and infection in this patient cohort was notably higher at 25.8% (8/31), representing a statistically significant increase compared to conventional surgical procedures. Furthermore, compromised blood supply subsequent to cavity necrosis contributes significantly to a reduced capacity for tissue healing. Within this cohort, two patients achieved complete wound healing after an extended period of intensive wound care, supplemented by the application of an adjacent skin flap to cover the wound. Conversely, one patient required additional surgery to manage a necrotic cavity, while another exhibited incomplete wound closure even after a 7-month follow-up. Notably, two patients experienced persistent, long-term pain following wound healing, necessitating prolonged oral analgesic use. This enduring discomfort may be attributed to the development of local fibrosis subsequent to radiation therapy.

The discussion on the role of preoperative neoadjuvant chemotherapy in sarcomas is currently a matter of ongoing debate within the field. The prevailing perspective posits that perioperative neoadjuvant chemotherapy holds the potential to effectively reduce tumor size, facilitate tumor downstaging, thereby aiding surgical resection (35). Furthermore, it is believed to eliminate satellite lesions and skip lesions surrounding the tumor, thereby contributing to an enhanced disease-free survival postoperatively (36, 37). The efficacy of chemotherapy is noted to be limited in low-grade malignant tumors, but it demonstrates effectiveness, particularly in high-risk sarcomas (11). Current research suggests that the application of preoperative neoadjuvant chemotherapy for high-risk soft tissue sarcomas may play a pivotal role in eradicating small lesions associated with distant metastases early in the disease course, ultimately leading to an improvement in patient survival (37).

The administration of a combination of multiple drugs before surgery, owing to the favorable tolerance observed in patients, serves as a viable strategy for rapidly reducing tumor activity and diminishing tumor volume (5). This approach is particularly advantageous in cases where tumors are situated in proximity to critical blood vessels and nerve sites, allowing for the feasibility of achieving an R0 resection (4). Our institution’s implementation of combined chemo-radiation therapy, in a preoperative sequential manner, demonstrated good tolerability, with a tumor downsizing rate (Objective Response Rate, ORR) of 32% (10/31), consistent with previously reported findings (13). Furthermore, our study revealed intriguing observations: while tumor volume increased in some patients, the solid component within the tumor exhibited a significant decrease, blood supply markedly reduced, and the final pathological results confirmed a tumor necrosis rate exceeding 90%. This phenomenon could be associated with the release of a substantial number of inflammatory mediators, leading to local edema subsequent to acute tumor necrosis or local tumor bleeding.

With the exception of one patient who experienced recurrence six months postoperatively, ultimately leading to upper limb amputation, all other patients in the preoperative sequential chemo-radiation group successfully underwent limb salvage. The primary complications associated with sequential chemo-radiation were bone marrow suppression and secondary fever. In this study, Significant chemotherapy toxicity occurred in 9/31 (29%) of patients. Following drug treatment, all patients demonstrated tolerance to the preoperative regimen. However, the cumulative impact of chemo-radiation exacerbated the extent of bone marrow suppression. To address this challenge, our hospital adopted the use of single-agent doxorubicin as a preoperative application for patients over 60 years old with a weaker constitution. This approach effectively mitigated the toxicity of chemotherapy and increased patient tolerance, facilitating the successful completion of preoperative medication.

Advantages of sequential chemo-radiation include the effective control of tumor growth and prevention of early metastasis (38). Clinical efficacy and pathological assessment guide the formulation of postoperative treatment measures and follow-up plans. Currently, this approach stands out as the most effective treatment method for high-risk soft tissue sarcomas. Large-segmented radiotherapy before surgery reduces the risk of tumor recurrence, shortens hospitalization time, and enhances patient compliance, resulting in economic benefits (33). Yet this technique should be circumvented in patients presenting with compromised integumentary integrity in the surgical zone, including those with localized infections, or in areas exhibiting extensive post-excisional cavities that are merely cloaked by skin, such as on the anterior aspect of the forearm and the lateral surface of the lower leg.

Pathological evaluation of tissue samples post preoperative chemoradiotherapy revealed an increased rate of tumor necrosis, with three instances exhibiting complete fibrosis within the lesion and pronounced intratumoral hemorrhage. Vacuolar degeneration was noted within the neoplastic cells. Subsequent examination of postoperative tissue specimens corroborated that the preoperative augmentation in tumor size was paralleled by a diminution in the viable tumor mass, attributable to necrosis and hemorrhage. Conversely, a decrease in tumor size prior to surgery may be linked to cellular necrosis, disintegration, and fibrotic transformation within the tumor (39, 40).

Yet, this study is retrospective, confined to a single institution, and encompasses a limited sample size, with a relatively brief duration of postoperative follow-up. Future investigations should extend to a larger cohort within the framework of a prospective observational study to validate these findings. The heterogeneity in chemotherapy protocols used may also affect the clinical outcomes. Moreover, the inclusion of patients with recurrent disease in this study may have led to an elevated rate of reoperation and associated complications.

## 5 Conclusion

In conclusion, the approach of preoperative sequential chemotherapy and radiotherapy combined with comprehensive surgical resection proves to be a practical and viable therapeutic strategy for high-risk soft tissue sarcomas. However, the increased incidence of bone marrow suppression and wound infection should be carefully managed. Further research is needed to validate these findings and optimize the treatment approach for soft tissue sarcomas.

## Data availability statement

The original contributions presented in the study are included in the article/supplementary material. Further inquiries can be directed to the corresponding author.

## Ethics statement

This study was approved by the Affiliated Cancer Hospital of Zhengzhou University (research protocol identification number: 2022–200-001). The studies were conducted in accordance with the local legislation and institutional requirements. The participants provided their written informed consent to participate in this study. Written informed consent was obtained from the individual(s) for the publication of any potentially identifiable images or data included in this article.

## Author contributions

GQ: Data curation, Investigation, Methodology, Writing – original draft. ZT: Investigation, Methodology, Project administration, Writing – review & editing. JW: Investigation, Methodology, Project administration, Writing – review & editing. CY: Investigation, Project administration, Writing – review & editing. XN: Investigation, Methodology, Resources, Writing – review & editing. WY: Data curation, Formal analysis, Investigation, Writing – review & editing.
